# 3D MR fingerprinting-derived myelin water fraction characterizing brain development and leukodystrophy

**DOI:** 10.1186/s12967-023-04788-y

**Published:** 2023-12-15

**Authors:** Hyun Gi Kim, Dongyeob Han, Jimin Kim, Jeong-Sun Choi, Kyung-Ok Cho

**Affiliations:** 1 Department of Radiology, Eunpyeong St. Mary’s Hospital, College of Medicine, The Catholic University of Korea, Seoul, Korea; 2 Siemens Healthineers Ltd., Seoul, Korea; 3 Department of Pharmacology, Department of Biomedicine & Health Sciences, Catholic Neuroscience Institute, Institute for Aging and Metabolic Diseases, College of Medicine, The Catholic University of Korea, 222 Banpo-daero, Seocho-Gu, Seoul, 06591 South Korea; 4 CMC Institute for Basic Medical Science, The Catholic Medical Center of The Catholic University of Korea, Seoul, Korea

**Keywords:** Magnetic resonance fingerprinting, Myelin water fraction, Proteolipid protein, Brain, Development, Children, Leukodystrophy, Pediatric

## Abstract

**Background:**

Magnetic resonance fingerprinting (MRF) enables fast myelin quantification via the myelin water fraction (MWF), offering a noninvasive method to assess brain development and disease. However, MRF-derived MWF lacks histological evaluation and remains unexamined in relation to leukodystrophy. This study aimed to access MRF-derived MWF through histology in mice and establish links between myelin, development, and leukodystrophy in mice and children, demonstrating its potential applicability in animal and human studies.

**Methods:**

3D MRF was performed on normal C57BL/6 mice with different ages, megalencephalic leukoencephalopathy with subcortical cyst 1 wild type (MLC1 WT, control) mice, and MLC 1 knock-out (MLC1 KO, leukodystrophy) mice using a 3 T MRI. MWF values were analyzed from 3D MRF data, and histological myelin quantification was carried out using immunohistochemistry to anti-proteolipid protein (PLP) in the corpus callosum and cortex. The associations between ‘MWF and PLP’ and ‘MWF and age’ were evaluated in C57BL/6 mice. MWF values were compared between MLC1 WT and MLC1 KO mice. MWF of normal developing children were retrospectively collected and the association between MWF and age was assessed.

**Results:**

In 35 C57BL/6 mice (age range; 3 weeks–48 weeks), MWF showed positive relations with PLP immunoreactivity in the corpus callosum (β = 0.0006, *P* = 0.04) and cortex (β = 0.0005, *P* = 0.006). In 12-week-old C57BL/6 mice MWF showed positive relations with PLP immunoreactivity (β = 0.0009, *P* = 0.003, R^2^ = 0.54). MWF in the corpus callosum (β = 0.0022, *P* < 0.001) and cortex (β = 0.0010, *P* < 0.001) showed positive relations with age. Seven MLC1 WT and 9 MLC1 KO mice showed different MWF values in the corpus callous (*P* < 0.001) and cortex (*P* < 0.001). A total of 81 children (median age, 126 months; range, 0–199 months) were evaluated and their MWF values according to age showed the best fit for the third-order regression model (adjusted R^2^ range, 0.44–0.94, *P* < 0.001).

**Conclusion:**

MWF demonstrated associations with histologic myelin quantity, age, and the presence of leukodystrophy, underscoring the potential of 3D MRF-derived MWF as a rapid and noninvasive quantitative indicator of brain myelin content in both mice and humans.

**Supplementary Information:**

The online version contains supplementary material available at 10.1186/s12967-023-04788-y.

## Background

Magnetic resonance fingerprinting (MRF) is a quantitative framework that can be used to assess brain development [1, 2]. With MRF, both T_1_ and T_2_ relaxation times can be acquired in a single acquisition within reasonable scanning times [3]. These relaxation time values reflect tissue properties that change as the brain matures [1]. In addition to T_1_ and T_2_ relaxation times, myelin content can be quantified with MRF [1]. MRF enables the measurement of myelin by separating myelin-bound water from other water components such as free water and intra- or extracellular water based on relaxation times [4]. The fraction of myelin-bound water in the total water pool is termed the myelin water fraction (MWF) [1, 5].

MWF characterizes brain development [1] because myelination is tightly linked to neural development [6, 7]. Conventional qualitative assessments of myelination using T_1_- and T_2_-weighted images [8] have evolved to quantitative assessments using diffusion tensor, relaxation times, or MWF [1, 6, 9]. A recent study reported on T_1_ and T_2_ relaxation times and MWF using 2-dimensional MRF from the developing brains of 28 children (age range, 0 to 5 years old) [1]. Another study focused on T_1_ and T_2_ relaxation times from the brain regions of 25 neonates (median-corrected gestational age, 263 days) according to age using 3-dimensional (3D) MRF [2]. Nevertheless, there is a notable lack of studies that histologically validate 3D MRF-derived MWF values with myelin markers.

Heritable white matter (WM) disorder is not uncommon with an estimated incidence of up to 1 per 8000 live births [10]. Brain magnetic resonance imaging (MRI) is a valuable assessment tool for WM disease in children [9, 11]. Still, studies that apply MRF-derived MWF to WM in leukodystrophies are limited. Megalencephalic leukoencephalopathy with subcortical cysts (MLC) is an inherited autosomal recessive disorder that shows infantile-onset cerebral WM edema that is characterized by myelin vacuolation [12, 13]. MLC patients develop macrocephaly during their first year of life, with conditions generally stabilizing afterward [13]. On brain MRI, diffuse swelling is noted in the cerebral WM with increased water content [13, 14]. Histologically, the brains of MLC patients show fluid-filled vacuoles within myelin sheaths [15] that likely affect quantified myelin expression in designated areas [16].

Since studies histologically validating 3D MRF-derived MWF values and corroborating MWF changes in leukodystrophy are currently lacking in literature, we aimed to histologically evaluate MRF-derived MWF and evaluate its associations with age and leukodystrophy in mice. In addition, since studies evaluating 3D MRF-derived MWF values in developing brains have only been performed on a limited number of children [1], we aimed to evaluate MWF and its associations with age in children, demonstrating the broad applicability of MRF studies in both mice and humans.

## Methods

### Animal study

#### Mice

Animal experiments were performed in compliance with the animal care guidelines issued by the National Institutes of Health and the Institutional Animal Use and Care Committee of The Catholic University of Korea. To evaluate myelination according to brain development, 35 C57BL/6 mice (19 females) were scanned from October 2021 to March 2022. The mice were of different ages from 3 to 48 weeks. To evaluate myelination in mice with leukodystrophy, 9 MLC1 wild type (WT, control model) mice and 9 MLC1 knock-out (KO, leukodystrophy model) mice were scanned from December 2021 to January 2022. Two MLC1 WT mice were excluded from the final MWF evaluation due to motion artifact of the images. Details on animal preparation are provided in Additional file 1: Appendix S1 and Additional file 3: Figure S1.

#### MRI Acquisitions and Postprocessing

**Fig. 1:**
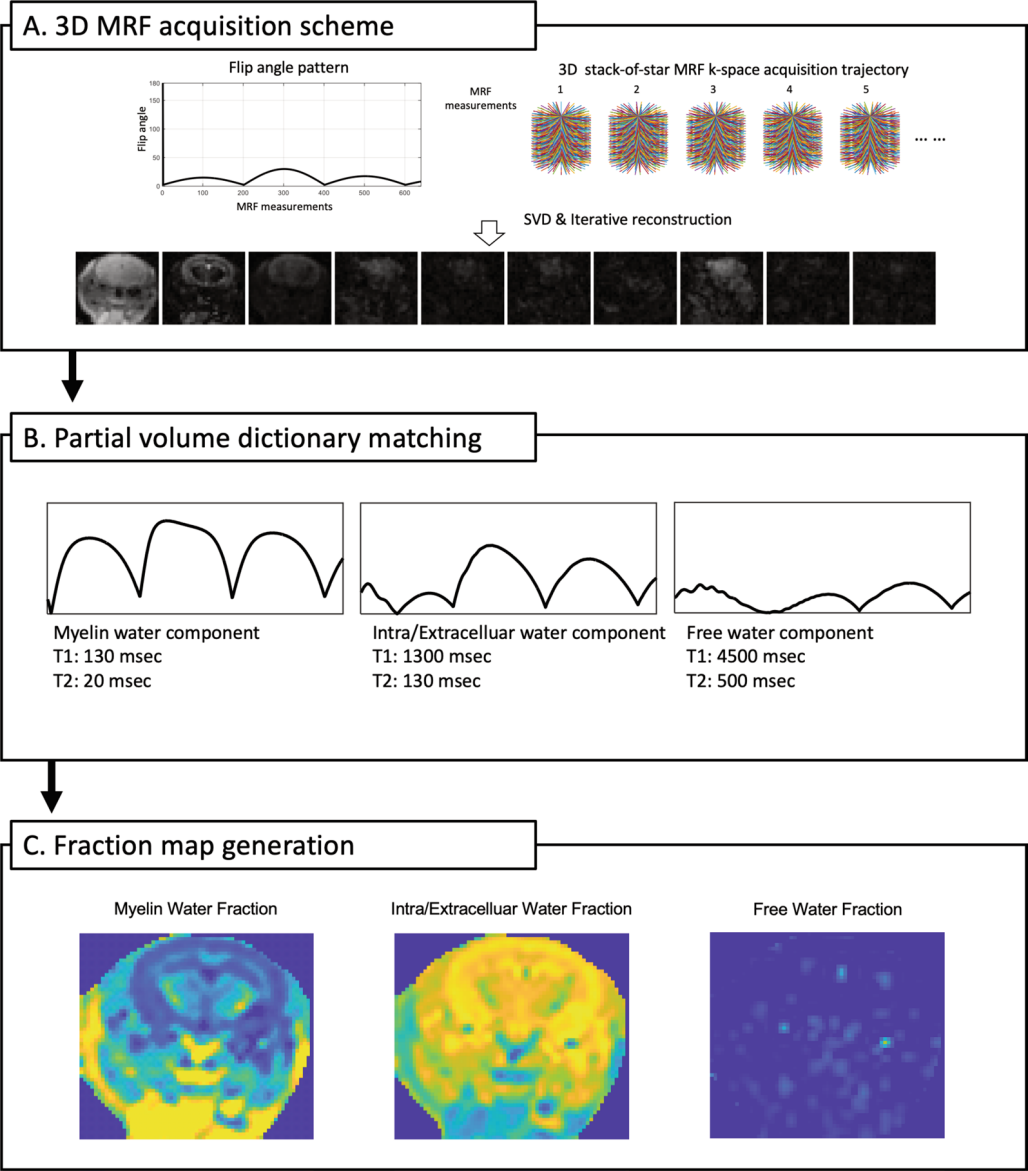
Flowchart of myelin water fraction (MWF) map generation using 3D MR fingerprinting (MRF). A 3D MRF acquisition scheme and reconstructed MRF images with a sinusoidal flip angle pattern and 3D stack-of-star acquisition trajectory (**A**). Signal evolutions of a partial volume dictionary for each component (myelin, intra/extracellular, and free water components) (**B**). Representative fraction maps of a mouse after dictionary matching (**C**). SVD, singular value decomposition

All images were acquired using a 3T MR scanner (Vida, Siemens Healthineers, Erlangen, Germany). In the animal study, a 6-channel birdcage coil (Stark Contrast, Erlangen, Germany) was used. 3D MRF with the stack-of-star acquisition was performed with the following parameters: repetition time, 10 ms; echo time, 4.84 ms; field of view, 60 × 60 × 24 mm^3^; voxel size, 0.5 × 0.5 × 2 mm^3^; flip angle, sinusoidal pattern; MRF time points, 640; number of radial spokes/MRF time points, 32; and acceleration factor along slice direction, 3 (scanning time: 17 min 55 sec) (Fig. 1). T_2_-weighted turbo spin echo scans for anatomical reference were performed with the following parameters: repetition time, 3000 ms; echo time, 64 ms; flip angle, 150 degrees; field of view, 42 × 42 mm^2^; resolution, 0.1 × 0.1 mm^2^; slice thickness, 1 mm; 18 slices acquired; grappa factor, 2; and averages, 8 (scanning time: 7 min 17 s).

T_1_ and T_2_ maps were derived from 3D MRF [2]. Then, a partial volume MRF analysis with a three-compartment model proposed in a previous study [1], was performed to measure MWF values. Predefined T_1_ and T_2_ values of each compartment were T_1_=130 ms, T_2_=20 ms for myelin water; T_1_=1300 ms, T_2_=130 ms for intracellular/extracellular water; T_1_=4500 ms, and T_2_=500 ms for free water (Fig. 1) [1, 4]. An iterative reconstruction method was also applied to improve image quality [2, 17]. 3D MRF utilized fast imaging with steady-state precession acquisition, and it did not include radiofrequency spoilers or spoiler gradients for complete spoiling. 3D MRF sequence detail and accuracy of T_1_ and T_2_ values derived from 3D MRF is described in prior studies [2, 17].

To assess changes in MWF in response to various predefined T_1_ and T_2_ values of myelin water, additional five different T_1_ and T_2_ combinations were applied to a 13-month-old MLC1 WT and a 13-month-old MLC1 KO mouse: combination 1, T_1_ = 10 ms, T_2_ = 10 ms; combination 2, T_1_ = 65 ms, T_2_ = 20 ms; combination 3, T_1_ = 130 ms, T_2_ = 10 ms; combination 4, T_1_ = 252 ms, T_2_ = 15 ms; combination 5, T_1_ = 828 ms, T_2_ = 72 ms. The predefined T_1_ and T_2_ values for intra/extracellular and free water were kept unchanged from the original settings.

#### Immunohistochemistry

Mice brain sections were incubated with mouse anti-proteolipid protein (PLP). Details for immunohistochemistry are provided in Additional file 1: Appendix S2. For immunoreactivity quantification, NIH ImageJ software was used (Additional file 1: Appendix S3).

#### Data analysis

One board-certified radiologist (H.G.K. with 14 years of experience in pediatric neuroradiology) drew regions of interest in the corpus callosum and cortex on mice MWF maps using T_2_-weighted images of each mouse as reference using ITK-SNAP (version 3.8.0; http://www.itksnap.org/) (Additional file 4: Figure S2). On brain sections with immunohistochemistry staining, one scientist (K.C. with 19 years of experience in mice brain study) drew regions of interest in the corpus callosum and cortex using NIH ImageJ.

### Children study

#### Children

**Fig. 2:**
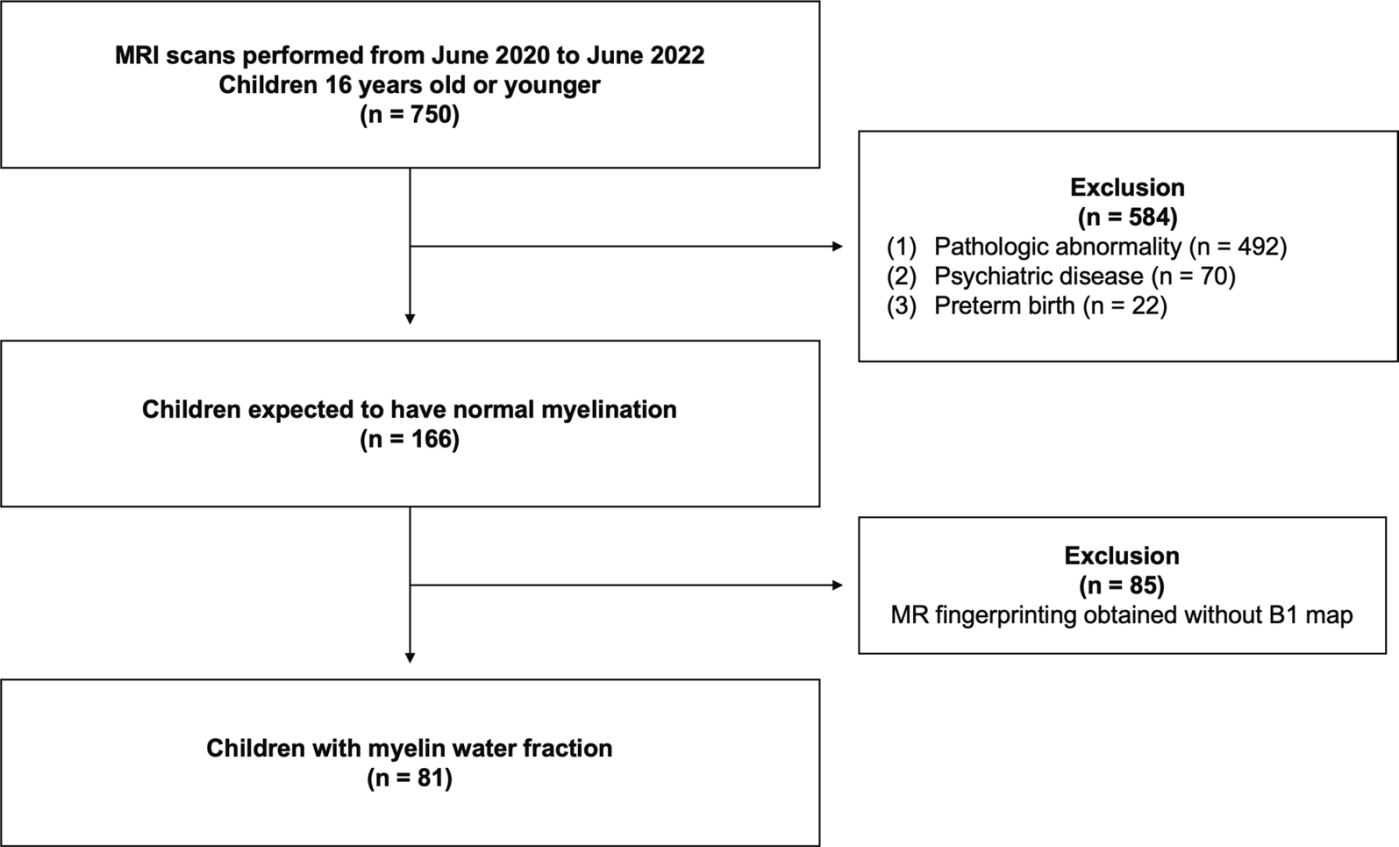
Flowchart of children selection

The Institutional Review Board approved this retrospective study of children, and the requirement for informed consent was waived. Our institution includes 3D MRF in routine clinical practice when brain MRI scans are performed. For neonates, we perform feed and wrap technique using a MedVac infant immobilizer (CFI Medical, USA) for MR scanning [18]. Clinical reports of MRI scans between June 2020 and June 2022 were consecutively reviewed, yielding 750 MRI studies. We excluded MRI exams for individuals with pathologic abnormalities, psychiatric diseases, or a history of preterm birth. Then, MRF obtained without a B_1_ map were excluded yielding 81 MRI studies (Fig. 2).

Children were divided by age into children 5 years or less and those older than 5 years. All MRI studies were reviewed and interpreted as having normal myelination by a board-certified pediatric radiologist (H.G.K. with 14 years of experience).

#### MRI Acquisitions and Postprocessing

In children, a 64-channel head and neck coil was used. 3D MRF with hybrid radial-EPI acquisition [2] was performed with the following parameters: repetition time, 7.7 ms; echo time, 4.84 ms; field of view, 256 × 256 × 144 mm^3^; voxel size, 0.7 × 0.7 × 2 mm^3^; flip angle, sinusoidal pattern; MRF time points, 640; number of radial spokes/MRF time points, 6; acceleration factor along slice direction, 5; and number of echo train length along slice direction; 4 (scanning time: 4 min 54 sec). T_1_, T_2_, and MWF maps were derived from 3D MRF, as detailed in the preceding section of this manuscript [1, 2].

#### Data Analysis

One board-certified radiologist (H.G.K. with 14 years of experience in pediatric neuroradiology) drew regions of interest on the frontal WM, parietal WM, occipital WM, posterior limb of the internal capsule, genu of the corpus callosum, splenium of the corpus callosum, caudate, putamen, and thalamus. Regions of interest in each brain region were drawn using T_1_ values maps as reference using ITK-SNAP (version 3.8.0; http://www.itksnap.org/) (Additional file 5: Figure S3).

#### Intra- and interobserver agreement

To evaluate the intraobserver agreement for MWF values in children, one board-certified radiologist (H.G.K. with 14 years of experience in pediatric neuroradiology) drew regions of interest in the brain regions at two-week intervals. To evaluate the interobserver agreement, two board-certified radiologists (J.K. and H.G.K. with 10 and 14 years of experience in neuroradiology and pediatric neuroradiology, respectively) blinded to clinical information independently drew the regions of interest.

### Age-matched animal and children

To evaluate the MWF values in the corpus callosum and cortex of age-matched groups, we selected C57BL/6 mice aged 3 weeks and children aged 12 years [19]. For the analysis of the children’s corpus callosum, the genu was selected as the region of interest.

### Statistics

Normality for variables was assessed using Kolmogorov–Smirnov test. Age and sex were compared between MLC1 WT and MLC1 KO mice using the Mann–Whitney test and Fisher’s exact test, respectively. Associations between ‘MWF and PLP immunoreactive area’ and ‘MWF and age’ were evaluated using linear regression in all C57BL/6 mice according to brain region of the corpus callosum and cortex. In 12-week C57BL/6 mice, association between ‘MWF and PLP immunoreactive area’ of both corpus callosum and cortex was evaluated using linear regression. To compare MWF values between MLC1 WT and MLC1 KO mice, the Mann–Whitney test was used. To compare PLP immunoreactive area values between MLC1 WT and MLC1 KO mice, the unpaired t-test was used. The age-MWF value relationships in children were analyzed with scatter plots and nonlinear regression. Second-order and third-order regressions were used for nonlinear regression and the regression models were compared. The age-T_1_ and age-T_2_ value relationships in children were analyzed with scatter plots and third-order regressions. β coefficients were derived to show the degree of change in MWF values or PLP immunoreactive areas (%) at 1-week intervals for C57BL/6 mice and at 1-year intervals for the children. Intraclass correlation coefficients (ICCs) were calculated to evaluate the intra- and interobserver agreement [20]. An ICC of 0.61–0.80 signified strong agreement and that of 0.81–1.00 signified to near complete agreement [21]. ICC was estimated based on a mean-rating (k = 2), absolute-agreement, two-way mixed-effects model. All statistical analyses were performed using software (SPSS version 29, SPSS; or GraphPad Prism version 8.4.2; GraphPad). All statistical analyses were performed by an author (H.G.K., 14 years of experience). Bonferroni correction was done for multiple testing and *P* values less than 0.05 were considered statistically significant.

## Results

### Animal study

#### Characteristics of the study sample

Thirty-five C57BL/6 mice of different ages (median age, 12 weeks; age range; 3–48 weeks) were evaluated: age of 3 weeks, 8 mice (6 females); 8 weeks, 8 mice (4 males); 12 weeks, 7 mice (4 males); 24 weeks, 5 mice (3 females); and 48 weeks, 7 mice (4 males). To compare myelination degree in mice with and without leukodystrophy, 9 MLC1 WT mice (median age, 17 months; age range, 12–20 months; 7 males) and 9 MLC1 KO mice (median age, 13 months; age range, 13–24 months; 6 females) were studies. For MWF study, the same 9 MLC1 KO mice and 7 MLC1 WT mice (median age, 17 months; age range, 12–20 months; 6 males) were evaluated. The demographic data of mice are shown in Table 1.

**Table 1:** Characteristics of the mice

Characteristic	C57BL/6	MLC1 WT	MLC1 KO	MLC1 WT vs MLC1 KO
	(n = 35)	(n = 9)	(n = 9)	*P* value
Age (weeks)	12 [16] (3–48)	68 [20] (48–80)	52 [24] (52–96)	0.80
Age (months)	3 [4] (1–12)	17 [5] (12–20)	13 [6] (13–24)	0.80
*Sex*				0.08
Male	16 (46)	7 (78)	3 (33)	
Female	19 (54)	2 (22)	6 (67)	

Data are presented as medians with IQRs in brackets and ranges in parentheses or numbers of patients with percentages in parentheses

MLC1 = megalencephalic leukoencephalopathy with subcortical cyst 1, WT = wild type, KO = knock-out

#### Association with histologic myelin staining and age

**Fig. 3:**
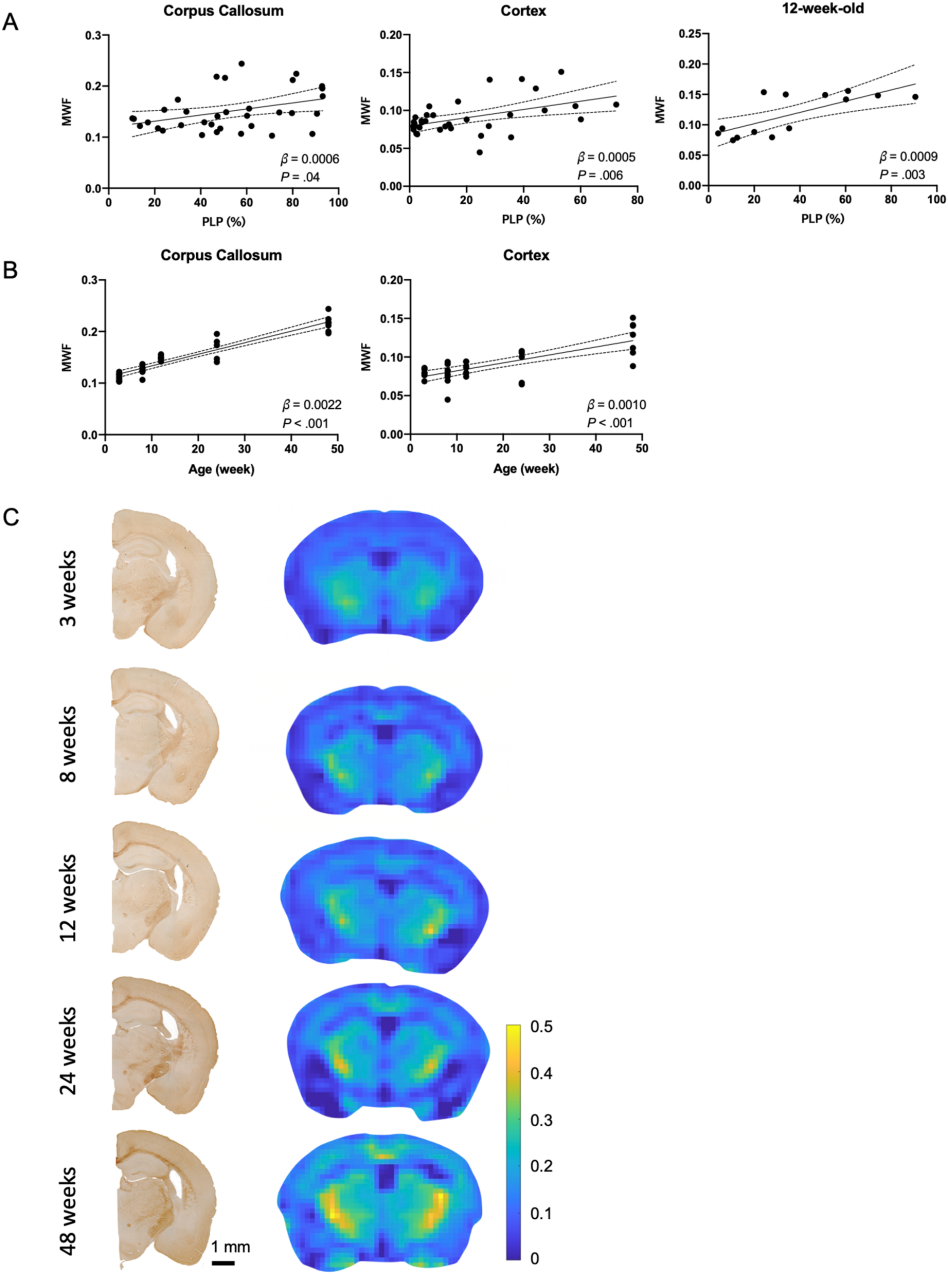
Myelin water fraction (MWF) according to the proteolipid protein (PLP) immunoreactive area value and age. MWF showed significant association with PLP immunoreactive area value in the corpus callosum (**A**, left) and cortex (**A**, middle) in all C57BL/6 mice and in seven 12-week-old C57BL/6 mice (**A**, right). MWF showed significant association with age in the corpus callosum (**B**, left) and cortex (**B**, right). Representative PLP immunoreactive staining images (left column) and MWF maps (right column) of C57BL/6 mice of different ages (**C**). Solid lines indicate the linear regression lines of best fit, and dashed lines indicate the 95% confidence intervals

In all C57BL/6 mice, MWF and PLP immunoreactive area values showed positive relationships in the corpus callosum (β = 0.0006, *P* = 0.04) and cortex (β = 0.0005, *P* = 0.006) (Fig. 3A). In 12-week C57BL/6 mice, MWF and PLP immunoreactive area values showed a positive relationship (β = 0.0009, *P* = 0.003, R^2^ = 0.54) (Fig. 3A). In C57BL/6 mice, the median [IQR] MWF values of the corpus callosum and cortex were 0.1 [0.06] and 0.08 [0.02], respectively. MWF values showed positive relationships with age in the corpus callosum (β = 0.0022, *P* < 0.001) and cortex (β = 0.0010, *P* < 0.001) (Fig. 3B). Representative MWF maps and PLP immunoreactive staining of C57BL/6 mice of different ages are shown in Fig. 3C. PLP immunoreactive area, MWF, T_1_, and T_2_ values for each age group are summarized in Additional file 2: Table S1.

#### Association with genetic white matter disease in mice

**Fig. 4:**
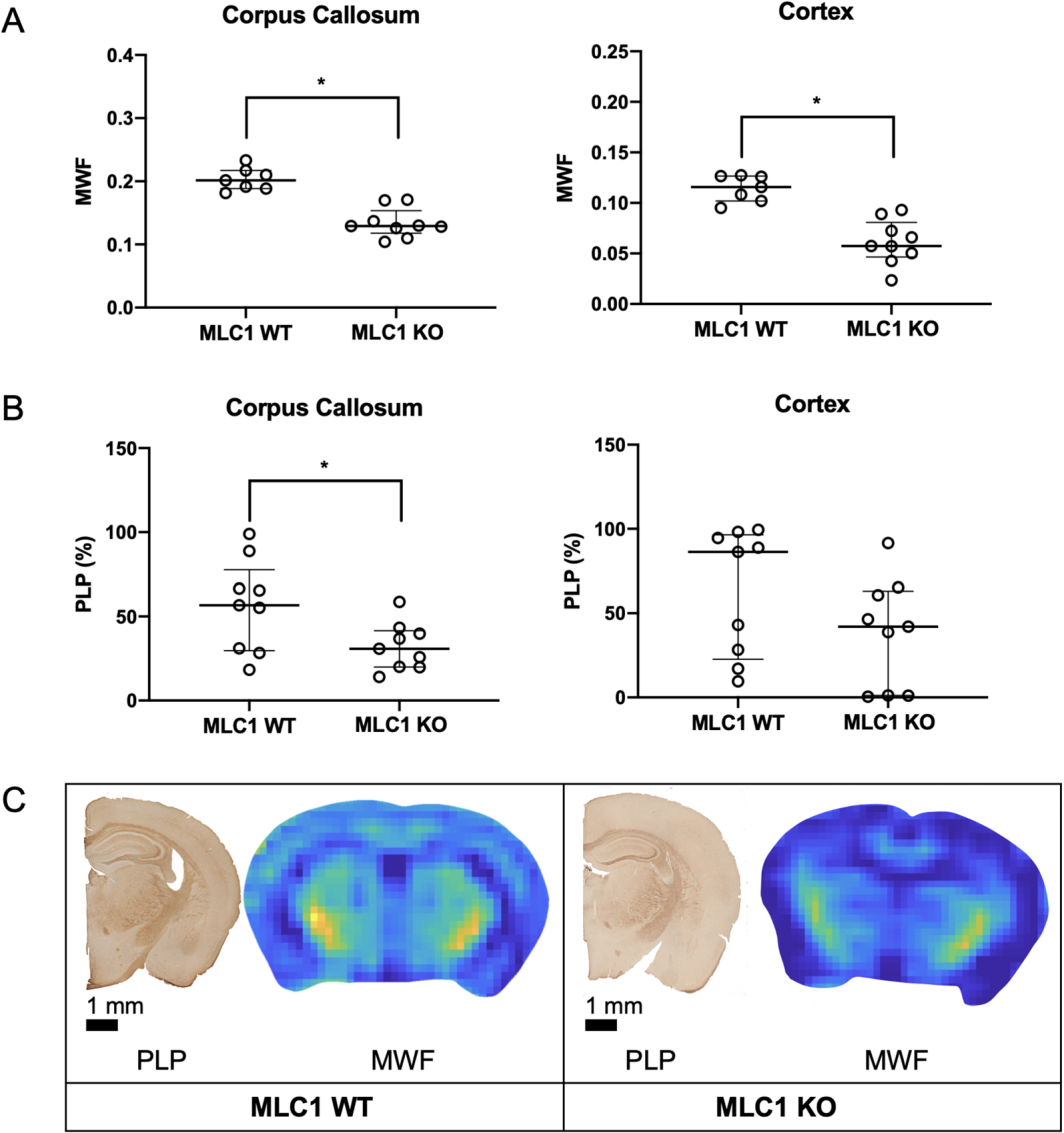
Comparison of myelin water fraction (MWF) and proteolipid protein (PLP) immunoreactive area values between leukodystrophy and control mice. Both the corpus callosum and cortex showed a difference in MWF values between MLC1 WT and KO mice (**A**). PLP immunoreactive area values was differed between MLC1 WT and KO mice in the corpus callous but not in the cortex (**B**). Representative PLP immunoreactive staining images and MWF maps of 13-month-old MLC1 WT and MLC1 KO mice (**C**). An asterisk (*) indicates a P-value smaller than 0.05

When MWF values were compared, both the corpus callosum (MLC1 WT vs MLC1 KO: 0.20 [IQR, 0.03] vs 0.13 [IQR, 0.04], respectively; *P* < 0.001) and cortex (MLC1 WT vs MLC1 KO: 0.12 [IQR, 0.02] vs 0.06 [IQR, 0.03], respectively; *P* < 0.001) showed a difference between MLC1 WT and KO mice (Table 2 and Fig. 4A). PLP immunoreactive area values was differed between MLC1 WT and KO mice in the corpus callous (MLC1 WT vs MLC1 KO: 57% [IQR, 50%] vs 31% [IQR, 22%], respectively; *P* = 0.03) but not in the cortex (MLC1 WT vs MLC1 KO: 86% [IQR, 74%] vs 42% [IQR, 62%], respectively; *P* = 0.16) (Table 2 and Fig. 4B). Representative MWF maps of MLC1 WT and KO mice are shown in Fig. 4C.

**Table 2:** Comparison of megalencephalic leukoencephalopathy subcortical cyst 1 wild type and knock-out mice

Parameter	MLC1 WT	MLC1 KO	*P* value
*Myelin water fraction*			
Corpus callosum	0.20 [0.03]	0.13 [0.04]	< 0.001
Cortex	0.12 [0.02]	0.06 [0.03]	< 0.001
*PLP (%)*			
Corpus callosum	57 [50]	31 [22]	0.03
Cortex	86 [74]	42 [62]	0.16

Data are presented as medians with IQRs in brackets and ranges in parentheses or numbers of mice with percentages in parentheses

MLC1 = megalencephalic leukoencephalopathy with subcortical cyst 1, WT = wild type, KO = knock-out, PLP = anti-proteolipid protein immunoreactive area

#### MWF according to predefined T_1_ and T_2_ values

Table 3 summarizes the MWF values derived from various predefined T_1_ and T_2_ combinations for myelin water. Consistent trends were observed across the different combinations: higher MWF values in the corpus callosum compared to the cortex, and higher values in MLC1 WT mice than in MLC1 KO mice. In detail, in Combination 1, where both T_1_ and T_2_ were set to 10 ms, lower MWF values were obtained compared to the original combination. In Combination 2, featuring a higher T_1_ than the original setting, reduced MWF values were observed. In contrast, Combination 3, with a lower T_2_ value, exhibited higher MWF values than the original. Combinations 4 and 5, both having higher T_1_ values but differing in T_2_ values (lower in Combination 4, higher in Combination 5), demonstrated increased MWF values compared to the original combination.

**Table 3:** Myelin water fraction values in megalencephalic leukoencephalopathy with subcortical cysts 1 wild-type and knock-out mice according to predefined T_1_ and T_2_ values of myelin water

	Combination		MLC1 WT mouse*		MLC1 KO mouse*	
	T_1_ (ms)	T_2_ (ms)	Corpus callosum	Cortex	Corpus callosum	Cortex
Original	130	20	0.178	0.123	0.121	0.021
1	10	10	0.022	0.006	0.008	0
2	65	20	0.101	0.057	0.058	0.001
3	130	10	0.221	0.158	0.159	0.037
4	252	15	0.312	0.256	0.243	0.130
5	828	72	0.888	0.837	0.837	0.574

^*^ A 13-month-old mouse, MLC1 = megalencephalic leukoencephalopathy with subcortical cyst 1, WT = wild type, KO = knock-out

### Children study

#### Characteristics of the study sample

A total of 81 children (median age, 126 months; age range, 0–199 months; 50 females) were evaluated. There were 57 children older than 5 years of age (median age, 151 months; age range, 68–199 months; 37 females). The demographic data of children are shown in Table 4.

**Table 4:** Characteristics of the children

Characteristic	Children (*n* = 81)
Age (months)	126 [144] (0–199)
*Sex*	
Male	31 (38)
Female	50 (62)

Data are presented as medians with IQRs in brackets and ranges in parentheses or numbers of patients with percentages in parentheses

#### Association with age in children

**Fig. 5:**
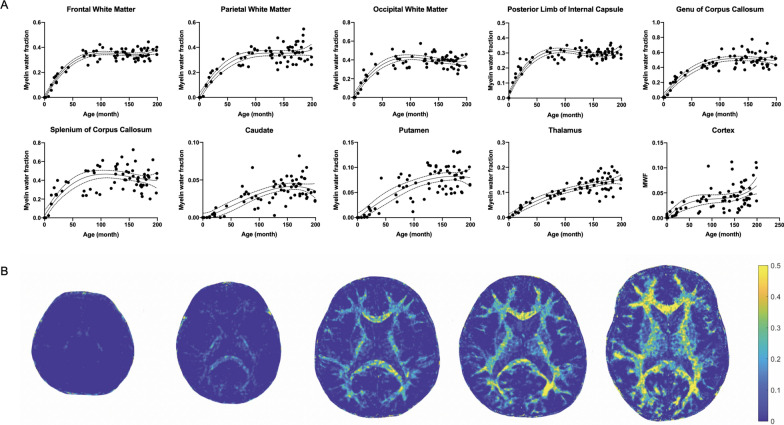
Myelin water fraction according to age in children. Scatter plots showing the myelin water fraction of multiple brain regions according to age in children (**A**). Representative axial MR fingerprinting-derived myelin water fraction maps of children of different ages (from left to right: a 2-month-old female, a 7-month-old female, a 20-month-old male, 42-month-old male, and 161-month-old male) (**B**). Solid lines indicate the third-order regression lines of best fit, and dashed lines indicate the 95% confidence intervals

MWF values for all children and those for the age subgroups are summarized in Additional file 2: Table S2. Median MWF values in the brain regions of all children ranged from 0.03 to 0.44. Median MWF value ranges in the brain regions of children 5 years old or less and children older than 5 years were 0–0.02 and 0.03–0.49, respectively. Scatter plots showed higher MWF values with age in the brain regions of children (Fig. 5A). Second- and third-order regressions demonstrated that MWF values were related to age in each brain region (Table 5). MWF values according to age was fitted to the third-order regression model (adjusted R^2^ range, 0.44 – 0.94, *P* < 0.001). When the two regression models were compared, frontal WM, parietal WM, occipital WM, posterior limb of the internal capsule, genu of the corpus callosum, and splenium of the corpus callosum showed the best fit with the third-order regression model (*P* value range; < 0.001 to 0.04). Representative MWF maps from children of different ages are shown in Fig. 5B. Scatter plots of T_1_ and T_2_ values according to age is shown in Additional file 6: Figure S4 and Additional file 7: Figure S5, and the third-order regression results are shown in Additional file 2: Table S3. T_1_ and T_2_ values of the brain regions according to age was fitted to the third-order regression model (T_1_, adjusted R^2^ range, 0.75−0.82, *P* < 0.001; T_2_, adjusted R^2^ range, 0.60−0.76, *P* < 0.001).

**Table 5:** Comparison of regression models assessing the relationships between age and myelin water fraction

Brain region	Second-order regression			Third-order regression			Second- vs Third-order regression
	Adjusted R^2^	RMSE	*P* value	Adjusted R^2^	RMSE	*P* Value	*P* value
Frontal white matter	0.90	0.04	< 0.001	0.94	0.03	< 0.001	< 0.001
Parietal white matter	0.83	0.06	< 0.001	0.85	0.06	< 0.001	0.002
Occipital white matter	0.81	0.07	< 0.001	0.84	0.06	< 0.001	< 0.001
Posterior limb of the internal capsule	0.83	0.05	< 0.001	0.90	0.03	< 0.001	< 0.001
Genu of the corpus callosum	0.86	0.08	< 0.001	0.86	0.08	< 0.001	0.04
Splenium of the corpus callosum	0.68	0.11	< 0.001	0.69	0.10	< 0.001	< 0.001
Caudate	0.64	0.01	< 0.001	0.65	0.01	< 0.001	0.19
Putamen	0.70	0.02	< 0.001	0.70	0.02	< 0.001	0.88
Thalamus	0.83	0.02	< 0.001	0.83	0.02	< 0.001	0.39
Cortex	0.42	0.02	< 0.001	0.44	0.02	< 0.001	0.06

RMSE = root mean squared error

#### Intra- and interobserver agreement

Intraobserver agreement for MWF was strong to near complete with the ICC ranging from 0.71 (95% CI: 0.55, 0.81) to 0.99 (95% CI: 0.99, 0.99) depending on the brain region. Interobserver agreement for MWF was also strong to near complete with the ICC ranging from 0.71 (95% CI: 0.55, 0.81) to 0.95 (95% CI: 0.92, 0.97). Intra- and interobserver agreement for MWF, T_1_, and T_2_ values are summarized in Additional file 2: Table S4.

### Age-matched animal and children

There were 8 C57BL/6 mice at 3 weeks of age and 5 children aged 12 years. The median [IQR] MWF of the corpus callosum was 0.11 [0.01] in the 3-week-old mice and 0.53 [0.15] in the 12-year-old children. For the cortex, the median [IQR] MWF was 0.08 [0.01] and 0.05 [0.03] in the 3-week-old mice and 12-year-old children, respectively.

## Discussion

M﻿yelin﻿﻿ quantification with brain MRI is important for the evaluation of normal brain development and leukodystrophy. MWF is considered an important parameter when quantifying myelin, but MWF derived from MRF has not been validated histologically or in a large study sample. In our study, we evaluated naive mice of different ages and transgenic mice showing leukodystrophy. 3D MRF-derived MWF values in the corpus callosum and cortex showed a positive relationship with histologic myelin immunoreactive areas and were higher with increasing age. Mice with leukodystrophy showed lower MWF values in the corpus callosum and cortex compared to control mice. In 81 normally developing children, the MWF values exhibited the anticipated developmental trend of myelin, aligning best with third-order regression models.

We histologically evaluated 3D MRF-derived MWF in mice of different ages and mice with leukodystrophy. The histologic evaluation of MWF imaging is an important step before MWF can be incorporated into actual preclinical and clinical research. MWF values in both animal models and humans have been measured to show their association with demyelinating conditions [22, 23] or autism spectrum disorder [24, 25]. MWF can be derived using the T_2_, T_2_*, T_1_, or steady-state-based MR sequences [5]. Compared to these MR sequences, 3D MRF allows a faster and high spatial resolution coverage of the brain in which whole brain coverage is possible in less than 10 min [2]. We used 3D MRF based on hybrid radial-interleaved EPI acquisition. In a past study, 2D synthetic MRI that quantified relaxation times and proton density by the multi-echo acquisition of a saturation recovery using turbo spin-echo readout (QRAPMASTER) was histologically evaluated for myelin quantification [26]. Synthetic MRI-derived myelin quantity was correlated with Luxol fast blue staining (spearman correlation coefficient = 0.74%, R^2^ = 0.55) [26]. This finding is consistent with our study, where MRF-derived MWF showed a relationship with PLP immunoreactivity (linear regression correlation coefficient = 0.0009, R^2^ = 0.54). The past study showed mean white and gray matter myelin values of 0.31 and 0.05, respectively [26]. The quantified values were consistent with our findings for children older than 5 years (median MWF: frontal WM, 0.35 and putamen, 0.08). However, direct comparison between our study and the past study is currently challenging since the myelin quantification derived from MRI is not highly specific to actual myelin density, with 45–46% of the signal unexplained by myelin density. Moreover, our study and the previous study employed different histological staining methods (PLP staining for our study versus Luxol fast blue staining for the synthetic MRI study). Given that different markers such as Luxol fast blue, PLP, myelin basic protein, and myelin oligodendrocyte glycoprotein can yield varying expression values following experimental demyelination and remyelination, despite generally reflecting myelination status [27], it will be interesting to identify the best and/or appropriate combination of markers tailored to the specific circumstances of myelin dynamics. Thus, while our histological evaluation of 3D MRF-derived MWF in mice highlights its promising application in research, the potential for direct comparison with previous studies is constrained by differences in specificity and staining methods, warranting additional investigation.

MWF values for both mice and children were generally higher according to age in our study, and the results are in line with previous studies [28, 29]. We showed that 3D MRF-derived MWF values can be used to assess brain maturation by showing quantitative myelination values. In the past, a qualitative assessment was generally used as the brain follows typical spatial developing patterns. However, now more quantitative [2, 6] and automatic assessment techniques [30] are available for measuring brain maturation. Age-related changes to myelin and its quantity have been studied in both children and adults [1, 2, 6, 29, 31]. In vivo evaluations of children’s MRI-based myelination quantification are mostly conducted by fitting the values into nonlinear equations [6, 32]. Although the developmental tendency aligned well with the trend of children's brain myelination in our study, the small MWF values in the subcortical and cortical regions (0.03–0.10) may merit a discussion regarding the accuracy of these values. Specifically, in the cortex, measurements can be inaccurate due to partial volume averaging effects, as the structure is relatively thin to measure, and the spatial resolution of 3D MRF may still be limited. However, since MR quantitative parameters showing abnormal brain maturation in patients with autism spectrum disorder [24, 25] or in children born preterm [33, 34] were revealed, it is worth having the relative degree of brain maturation in children assessed by 3D MRF.

Beyond MRF and synthetic MRI, a spectrum of MR techniques exists for quantifying myelination, including ultra-short echo-time (UTE), magnetization transfer (MT), inhomogeneous magnetization transfer (ihMT), and quantitative susceptibility mapping (QSM) [35]. Among these modalities, ihMT and QSM have demonstrated very strong correlations with myelination in animal models (R^2^ = 0.85 − 0.94), and UTE and MT techniques have exhibited strong correlations (R^2^ = 0.51 − 0.60) [35]. Our study showed a strong correlation between MWF and PLP staining (R^2^ = 0.54), aligning with prior studies utilizing MWF (R^2^ = 0.55) [35]. Contrary to ihMT and QSM, regarded as indirect quantitative MRI strategies for myelination mapping, MRF is classified as a rapid, direct multiparametric quantitative MRI approach, employing imaging data to directly synthesize parameter maps [36]. MRF facilitates the generation of T_1_ and T_2_ maps, as well as MWF maps, within a reduced time frame, whereas indirect myelination quantification methods necessitate extended scan durations and provide a restricted parameter set. Given the importance of fast scanning, especially for neonates, we employed MRF in the current study. However, other promising MRI methods such as ihMT and QSM will undoubtedly complement the accurate analysis of myelination in the brain.

In our study, MLC1 KO (leukodystrophy model) mice showed lower MWF values compared to MLC1 WT (control model) mice. This trend remained consistent across varying predefined T_1_ and T_2_ values for myelin water. MLC is an inheritable disorder characterized by cerebral white matter edema [37]. Histologically, brain content increases and intramyelinic vacuoles are observed with MLC [37]. This histologic alteration in mice can be seen as early as in 3 months of age and becomes prominent at 7–12 months old [38]. When we evaluated myelination of MLC mice, cortical MWF significantly differed between MLC1 WT and MLC1 KO mice, while PLP staining showed a reducing trend in MLC1 KO mice. As both MWF and PLP results assessed from the corpus callosum demonstrated a significant reduction by MLC1 deletion, this may suggest that MWF is more sensitive to show changes due to leukodystrophy, however, a larger number of samples with different ages would be needed to confirm this assumption. In addition, since the genetic type of MLC is related to its clinical presentation and prognosis [39], MWF assessment can potentially be used for genetic subtype classification.

Age-matched mice and children’s corpus callosum and cortex MWF showed similar trends in both species with values for corpus callosum (mice, 0.11; children 0.53) and cortex (mice, 0.08; children 0.05). The comparative literature on myelination using MRI methodologies between murine and human models is scarce. This scarcity might be attributable to the technical difficulties in standardizing MRI sequences for both species. Murine neuroimaging commonly employs high-field MRI scanners (7 T or higher), whereas human neuroimaging studies generally utilize lower filed scanners (3 T or lower). There was one study showing the myelination trajectories in canines and simians using T_2_ relaxation time with a 2.35 T MRI scanner [40], suggesting a potential for cross-species applicability. However, the study did not directly compare the quantified myelination between species, possibly reflecting more on the brain's maturation stage than a species-specific difference.

Our study has several limitations. First, a three-pool model was used to calculate MWF from 3D MRF based on previous studies [1, 4]. The T_1_ and T_2_ values that compared the water pools were based on prior assumptions and calculations in adults and children [1, 4]. However, our MWF values were higher than prior MWF studies [41] and the accuracy of the MWF may be influenced by these predefined values as it is shown from our results (Table 3). Future investigation should consider curating specific T_1_ and T_2_ values tailored to children and adults. In addition, although we assumed that differences in relaxation time between compartments would not be drastically different for mice and humans, it is still debatable whether the same modeling can be applied to animal studies. Second, the selection of age ranges for mice and children differs, making direct comparisons between the two species in specific developmental periods challenging. The oldest C57BL/6 mice in our study were 48 weeks old, equivalent to 38–47 human years [19]. In contrast, our human data focused on evaluating MWF in children up to 16 years old. It would be interesting to explore whether our MWF algorithm can also be applied to older humans in future studies. Third, data were collected retrospectively and some of the children may have had factors affecting myelination. To lower this possibility, we showed MWF changes according to age, which fit with typical developing patterns. We also excluded children with structural abnormalities or with medical histories that could alter the course of normal development. However, the retrospective nature of this study makes it difficult to conclude that the children were healthy in every aspect. Fourth, considering the wide range of children, including young ones prone to motion artifacts, there is a possibility that MWF values might be affected by movement during MRI scanning. To reduce motion and increase safety, we applied the feed-and-wrap technique for neonates. However, for other children, whether non-sedated or sedated, no specific method was employed to mitigate motion. Completely eliminating motion during MRI scans is challenging, and such movement can impact MWF values. Nevertheless, MRF has shown robustness to motion in prior studies [42]. The influence of motion on MWF parameters derived from MRF warrants future investigation.

## Conclusions

In conclusion, we acquired MWF values using 3D MRF in mice of different ages, mice with leukodystrophy, and children of different ages. MWF values from 3D MRF were in high agreement with the values obtained from histopathologic myelin staining. We observed higher MWF values with increasing age in both mice and children. MWF values were different between mice with and without leukodystrophy. Therefore, MWF derived from 3D MRF can be a promising parameter of myelin degree in the brain that can be attained rapidly and noninvasively in both mice and humans. To establish MWF as a quantitative diagnostic tool, future studies for defining accurate T_1_ and T_2_ values for MWF measurement for both mice and humans are necessary. Longitudinal studies utilizing MWF to evaluate normal and pathological brain development will further enhance its role as a prognostic and monitoring marker in patients with diseases.

### Supplementary Information

**Additional file 1: Appendix S1.** Animal preparation. **Appendix S2.** Immunohistochemistry of proteolipid protein (PLP). **Appendix S3.** Quantitative microscopic analysis.

**Additional file 2: Table S1.** Summary of sex, myelin basic protein immunoreactive area, myelin water fraction, T_1_, and T_2_ values in age groups of C57BL/6 mice. **Table S2.** Myelin water fraction values in each brain region in children. **Table S3.** Third-order regression models assessing the relationships between age and relaxometry values. **Table S4.** Intra- and interobserver agreement of myelin water fraction, T_1_, and T_2_ values in each brain region.

**Additional file 3: Figure S1.** MLC1 immunohistochemistry and expression in astrocytes (A, B) of MLC1 WT and KO Mice. Regions indicated by white squares are magnified in the far-right column. MLC1 deletion is verified by western blotting, and MLC1 expression (C) is compared between MLC1 WT and KO mice. Three asterisks (***) indicate a *P* value smaller than 0.001.

**Additional file 4: Figure S2.** Regions of interest drawn in the cortex and corpus callosum in a mouse on the T_2_-weighted image (left) and myelin water fraction map (right).

**Additional file 5: Figure S3.** Regions of interest drawn in multiple brain regions in a child using T_1_ value map. WM = white matter, CC = corpus callosum.

**Additional file 6: Figure S4.** Scatter plots of T_1_ values in children according to age. Solid lines indicate the third-order regression lines of best fit, and dashed lines indicate the 95% confidence intervals.

**Additional file 7: Figure S5.** Scatter plots of T_2_ values in children according to age. Solid lines indicate the third-order regression lines of best fit, and dashed lines indicate the 95% confidence intervals.

## Data Availability

All data generated or analyzed during this study were included either in this article methods section. Other data supporting the findings of this study are available from the corresponding author upon reasonable request.

## Abbreviations

ihMT: Inhomogeneous magnetization transfer
KO: Knock-out
MRF: Magnetic resonance fingerprinting
MRI: Magnetic resonance imaging
MLC: Megalencephalic leukoencephalopathy with subcortical cysts
MT: Magnetization transfer
MWF: Myelin water fraction
QSM: Quantitative susceptibility mapping
UTE: Ultra-short echo-time
WT: Wild type
WM: White matter
3D: 3-Dimensional
