# Supplementary material for: 3D MR fingerprinting-derived myelin water fraction characterizing brain development and leukodystrophy
Source: J Transl Med. 2023 Dec 15;21:914. doi: 10.1186/s12967-023-04788-y (PMC10725020; doi:10.1186/s12967-023-04788-y)
Supplement: Supplementary file 1 — Additional file 1: Appendix S1. Animal preparation. Appendix S2. Immunohistochemistry of proteolipid protein (PLP). Appendix S3. Quantitative microscopic analysis. [file 12967_2023_4788_MOESM1_ESM.docx]

**Additional Materials**

Appendix S1. Animal preparation

MLC1 wild type (WT) and knock-out (KO) mice were generated using the FAST (Flexible Accelerated STOP TetO-knockin) system (1). We purchased both MLC1 STOP TetO-knockin and MLC1 TetO-knockin mice from RIKEN BRC and crossed them with littermates to generate MLC1 KO and WT mice, respectively. Mice were genotyped by the polymerase chain reaction (PCR) using genomic DNA and primers for the knockin band (5’-AGGGAATGGTGGTCTGAGTCTGTT-3’, 5’-CTCGACCCGGGTACCGAGCTC-3’) and WT (5’-AGGGAATGGTGGTCTGAGTCTGTT-3’, 5’- GAGAACACCCATGTCTTGTAGCTG-3’).

To confirm MLC1 deletion, western blotting was performed (2) (Supplementary Figure 1). The hippocampus was lysed in ProEX™ CETi lysis buffer (TransLab Biosciences). Lysates were centrifuged, and protein was extracted from the supernatant. Equal amounts of protein (30 µg) were separated onto 12% SDS-polyacrylamide gels and then transferred to polyvinylidene difluoride membranes. After washing the membranes with Tris-buffered saline with 0.1% Tween-20 (TBST), they were incubated with 5% bovine serum albumin (BSA) in TBST for an hour at room temperature. The membranes were then placed in anti-MLC1 (1:1,000, Abcam, AB130770) antibodies in a shaker at 4℃ overnight. The next day, after another round of washing, horseradish peroxidase-conjugated secondary anti-rabbit IgG (1:2,000, GeneTex) was added to the membranes at room temperature for 2 h. Afterward, bands were detected by the enhanced chemiluminescence detection kit (Elpis Biotech, Inc.). For loading controls, the membranes were stripped with Restore Western Blot Stripping buffer (Thermo Scientific) and re-probed with mouse monoclonal anti-glyceraldehyde-3-phosphate dehydrogenase (GAPDH, 1:1,000, Santa Cruz Biotechnology, Inc., SC-32233). The membranes were incubated with horseradish peroxidase-conjugated secondary antibodies (1:2000, GeneTex) and visualized under ImageQuant LAS 4000 (Fujifilm). Finally, relative quantification of immunoreactivity was carried out for each band using densitometric analysis. A statistical analysis of MLC1 expression was performed using the unpaired Student’s t-test as normal distribution was assumed. Specifically, 60 kDa of MLC1 was analyzed using the unpaired t-test with Welch’s correction and 30 kDa of MLC1 was assessed using the unpaired t-test. All mice were housed at standard temperature (22 ± 2°C) in a light-controlled environment with free access to food and water.

Appendix S2. Immunohistochemistry of proteolipid protein (PLP)

Free-floating tissue sections were washed in 0.01 M PBS, followed by the blocking of nonspecific binding with 1% bovine serum albumin, 5% normal donkey serum, and 0.3% Triton X-100 in PBS for 1 h at room temperature. Sections were then incubated with chicken anti-PLP (1:500, Genetex, GTX85465) for 3 days at 4°C. Then, after washing the sections with 0.01 M PBS three times, they were incubated with biotin-conjugated anti-chicken IgG (1:100, Jackson ImmunoResearch Laboratories, 103-065-155) or anti-mouse IgG (1:200, Vector Laboratories, PK6102) for 2 h at room temperature, respectively. Signals were amplified using the VECTASTAIN ABC kit (Vector Laboratories, PK6100) for 1 h at room temperature, followed by visualization with 0.05% diaminobenzidine tetrahydrochloride (DAB). Finally, sections were mounted with Canada Balsam and imaged under an upright microscope (BX51; Olympus). Half-brain images were created using the live stitch function of Mosaic 2.1 software (Tucsen). For double labeling, primary antibodies were incubated with rabbit anti-MLC1 (1:200, Abcam, ab130770) overnight at 4°C. Next day, after washing with 0.01 M PBS three times, the sections were incubated with a cy3-conjugated fluorescent secondary antibody (1:500, Jackson ImmunoResearch Laboratories, 711-165-152), followed by incubation with mouse anti-GFAP (1:500, Millipore, MAB360) overnight at 4°C. On the third day, sections were incubated with Alexa Fluor 488-conjugated anti-mouse IgG (1:500, Jackson ImmunoResearch Laboratories, 115-545-205) for 2 h at room temperature. Finally, sections were mounted on the VECTASHIELD Hardset Antifade Mounting Medium with DAPI (Vector Laboratories, H150010144) and assessed under a confocal microscope (LSM700; Carl Zeiss Microscopy).

Appendix S3. Quantitative microscopic analysis

PLP immunoreactivity was quantitatively analyzed using NIH ImageJ software. The pixel area of the corpus callosum and cortex was measured to define the region of interest and the pixel intensity of the non-tissue area was measured as the background intensity. Using the threshold function, the area of pixels greater than the background intensity was measured as the PLP staining area. Data were reported as the percentage of the PLP staining area based on the area of the corpus callosum and cortex.

1. Tanaka KF, Ahmari SE, Leonardo ED, et al. Flexible Accelerated STOP Tetracycline Operator-knockin (FAST): a versatile and efficient new gene modulating system. Biol Psychiatry 2010;67(8):770-773.
2. Choi IY, Shim JH, Kim MH, et al. Truncated Neogenin Promotes Hippocampal Neuronal Death after Acute Seizure. Neuroscience 2021;470:78-87.
