# Supplementary material for: 3D MR fingerprinting-derived myelin water fraction characterizing brain development and leukodystrophy
Source: J Transl Med. 2023 Dec 15;21:914. doi: 10.1186/s12967-023-04788-y (PMC10725020; doi:10.1186/s12967-023-04788-y)
Supplement: Supplementary file 2 — Additional file 2: Table S1. Summary of sex, myelin basic protein immunoreactive area, myelin water fraction, T1, and T2 values in age groups of C57BL/6 mice. Table S2. Myelin water fraction values in each brain region in children. Table S3. Third-order regression models assessing the relationships between age and relaxometry values. Table S4. Intra- and interobserver agreement of myelin water fraction, T1, and T2 values in each brain region. [file 12967_2023_4788_MOESM2_ESM.docx]

**Table S1:** Summary of sex, myelin basic protein immunoreactive area, myelin water fraction, T_1_, and T_2_ values in age groups of C57BL/6 mice

| Parameters | 3 weeks (n = 8) | 8 weeks (n = 8) | 12 weeks (n = 7) | 24 weeks (n = 5) | 48 weeks (n = 7) |
| --- | --- | --- | --- | --- | --- |
| PLP (%) |  |  |  |  |  |
| Corpus callosum | 44 [33] (14 – 71) | 37 [46] (10 – 89) | 60 [40] (24 – 91) | 80 [54] (30 – 93) | 80 [42] (47 – 93) |
| Cortex | 2 [4] (1 – 15) | 3 [11] (1 – 25) | 13 [22] (4 – 35) | 36 [44] (7 – 73) | 44 [30] (17 – 60) |
| MWF |  |  |  |  |  |
| Corpus callosum | 0.11 [0.01] (0.10 – 0.12) | 0.13 [0.01] (0.11 – 0.14) | 0.15 [0.01] (0.14 – 0.16) | 0.17 [0.04] (0.14 – 0.20) | 0.22 [0.02] (0.20 – 0.24) |
| Cortex | 0.08 [0.01] (0.07 – 0.09) | 0.08 [0.02] (0.04 – 0.09) | 0.09 [0.02] (0.07 – 0.09) | 0.10 [0.04] (0.06 – 0.11) | 0.13 [0.04] (0.09 – 0.15) |
| T_1_ (ms) |  |  |  |  |  |
| Corpus callosum | 1131 [19] (1114 – 1159) | 1116 [15] (1107 – 1140) | 1098 [27] (1076 – 1128) | 1049 [43] (1043 – 1109) | 979 [58] (945 – 1041) |
| Cortex | 1197 [25] (1169 – 1212) | 1212 [36] (1189 – 1246) | 1207 [22] (1193 – 1225) | 1192 [56] (1156 – 1250) | 1139 [83] (1074 – 1203) |
| T_2_ (ms) |  |  |  |  |  |
| Corpus callosum | 34 [7] (26 – 38) | 27 [6] (25 – 45) | 26 [3] (23 – 35) | 24 [17] (18 – 43) | 24 [4] (16 – 27) |
| Cortex | 34 [6] (26 – 39) | 27 [4] (25 – 46) | 27 [4] (23 – 33) | 25 [16] (20 – 45) | 25 [5] (20 – 29) |

Note.—Data are presented as medians with IQRs in brackets and ranges in parentheses, or numbers of patients with percentages in parentheses.

MWF = Myelin water fraction, PLP = anti-proteolipid protein immunoreactive area.

**Table S2: Myelin water fraction values in each brain region in children**

| Brain region | Total (n = 81) | 5 years or less (n = 24) | Older than 5 years (n = 57) |
| --- | --- | --- | --- |
| Frontal white matter | 0.33 [0.19] | 0.01 × 10^-2^ [0.15] | 0.35 [0.06] |
| Parietal white matter | 0.31 [0.18] | 0.01 × 10^-2^ [0.15] | 0.35 [0.09] |
| Occipital white matter | 0.37 [0.17] | 0.04 × 10^-2^ [0.22] | 0.39 [0.09] |
| Posterior limb of the internal capsule | 0.28 [0.09] | 0.02 × 10^0^ [0.20] | 0.30 [0.04] |
| Genu of the corpus callosum | 0.44 [0.25] | 0.34 × 10^-2^ [0.23] | 0.49 [0.11] |
| Splenium of the corpus callosum | 0.38 [0.23] | 0.04 × 10^-2^ [0.25] | 0.43 [0.17] |
| Caudate | 0.03 [0.04] | 0 [0.00] | 0.03 [0.02] |
| Putamen | 0.06 [0.08] | 0 [0.01] | 0.08 [0.04] |
| Thalamus | 0.10 [0.10] | 0.04 × 10^-3^ [0.04] | 0.12 [0.05] |
| Cortex | 0.03 [0.03] | 0.04 × 10^-1^ [0.01] | 0.04 [0.03] |

Note.—Data are presented as medians with IQRs in brackets.

**Table S3: Third-order regression models assessing the relationships between age and relaxometry values**

|  | T_1_ value | | | T_2_ value | | |
| --- | --- | --- | --- | --- | --- | --- |
|  |  |  |  |  |  |  |
| Brain region | Adjusted R^2^ | RMSE | *P* Value | Adjusted R^2^ | RMSE | *P* Value |
| Frontal white matter | 0.80 | 308 | <.001 | 0.73 | 24 | <.001 |
| Parietal white matter | 0.77 | 317 | <.001 | 0.71 | 28 | <.001 |
| Occipital white matter | 0.77 | 300 | <.001 | 0.73 | 18 | <.001 |
| Posterior limb of the internal capsule | 0.78 | 146 | <.001 | 0.70 | 10 | <.001 |
| Genu of the corpus callosum | 0.77 | 304 | <.001 | 0.67 | 23 | <.001 |
| Splenium of the corpus callosum | 0.77 | 230 | <.001 | 0.68 | 13 | <.001 |
| Caudate | 0.75 | 135 | <.001 | 0.68 | 10 | <.001 |
| Putamen | 0.81 | 108 | <.001 | 0.73 | 8 | <.001 |
| Thalamus | 0.82 | 157 | <.001 | 0.76 | 10 | <.001 |
| Cortex | 0.70 | 158 | <.001 | 0.60 | 11 | <.001 |

RMSE = root mean squared error

**Table S4: Intra- and interobserver agreement of myelin water fraction, T_1_, and T_2_ values in each brain region**

|  | MWF | | | T_1_ | | | T_2_ | | |
| --- | --- | --- | --- | --- | --- | --- | --- | --- | --- |
|  | ICC | 95% CI | | ICC | 95% CI | | ICC | 95% CI | |
| Intraobserver agreement |  |  | |  |  | |  |  | |
| Frontal white matter | 0.98 | 0.97 | 0.99 | 0.95 | 0.92 | 0.97 | 0.95 | 0.93 | 0.97 |
| Parietal white matter | 0.97 | 0.95 | 0.98 | 0.95 | 0.93 | 0.97 | 0.96 | 0.93 | 0.97 |
| Occipital white matter | 0.95 | 0.92 | 0.97 | 0.94 | 0.91 | 0.96 | 0.93 | 0.88 | 0.95 |
| Posterior limb of internal capsule | 0.99 | 0.99 | 0.99 | 0.95 | 0.92 | 0.97 | 0.94 | 0.91 | 0.96 |
| Genu of corpus callosum | 0.97 | 0.95 | 0.98 | 0.96 | 0.93 | 0.97 | 0.95 | 0.93 | 0.97 |
| Splenium of corpus callosum | 0.91 | 0.86 | 0.94 | 0.94 | 0.90 | 0.96 | 0.91 | 0.86 | 0.94 |
| Caudate | 0.89 | 0.83 | 0.93 | 0.96 | 0.88 | 0.95 | 0.93 | 0.89 | 0.95 |
| Putamen | 0.87 | 0.79 | 0.91 | 0.91 | 0.85 | 0.94 | 0.92 | 0.87 | 0.95 |
| Thalamus | 0.98 | 0.96 | 0.98 | 0.95 | 0.92 | 0.97 | 0.95 | 0.93 | 0.97 |
| Cortex | 0.71 | 0.55 | 0.81 | 0.88 | 0.81 | 0.92 | 0.90 | 0.84 | 0.94 |
| Interobserver agreement |  |  |  |  |  |  |  |  |  |
| Frontal white matter | 0.92 | 0.88 | 0.95 | 0.95 | 0.92 | 0.97 | 0.94 | 0.91 | 0.96 |
| Parietal white matter | 0.91 | 0.86 | 0.94 | 0.96 | 0.93 | 0.97 | 0.95 | 0.93 | 0.97 |
| Occipital white matter | 0.89 | 0.83 | 0.93 | 0.94 | 0.90 | 0.96 | 0.92 | 0.87 | 0.95 |
| Posterior limb of internal capsule | 0.95 | 0.92 | 0.97 | 0.94 | 0.91 | 0.96 | 0.94 | 0.90 | 0.96 |
| Genu of corpus callosum | 0.87 | 0.80 | 0.92 | 0.95 | 0.93 | 0.97 | 0.93 | 0.89 | 0.96 |
| Splenium of corpus callosum | 0.85 | 0.76 | 0.90 | 0.94 | 0.90 | 0.96 | 0.86 | 0.78 | 0.91 |
| Caudate | 0.81 | 0.70 | 0.88 | 0.92 | 0.88 | 0.95 | 0.92 | 0.88 | 0.95 |
| Putamen | 0.83 | 0.74 | 0.89 | 0.93 | 0.89 | 0.95 | 0.93 | 0.88 | 0.95 |
| Thalamus | 0.93 | 0.89 | 0.95 | 0.95 | 0.92 | 0.97 | 0.94 | 0.91 | 0.96 |
| Cortex | 0.71 | 0.55 | 0.81 | 0.88 | 0.81 | 0.92 | 0.89 | 0.83 | 0.93 |

ICC = Intraclass correlation coefficients, 95% IC = 95% confidence interval
