# Supplementary figures and images for: 3D MR fingerprinting-derived myelin water fraction characterizing brain development and leukodystrophy
Source: J Transl Med. 2023 Dec 15;21:914. doi: 10.1186/s12967-023-04788-y (PMC10725020; doi:10.1186/s12967-023-04788-y)

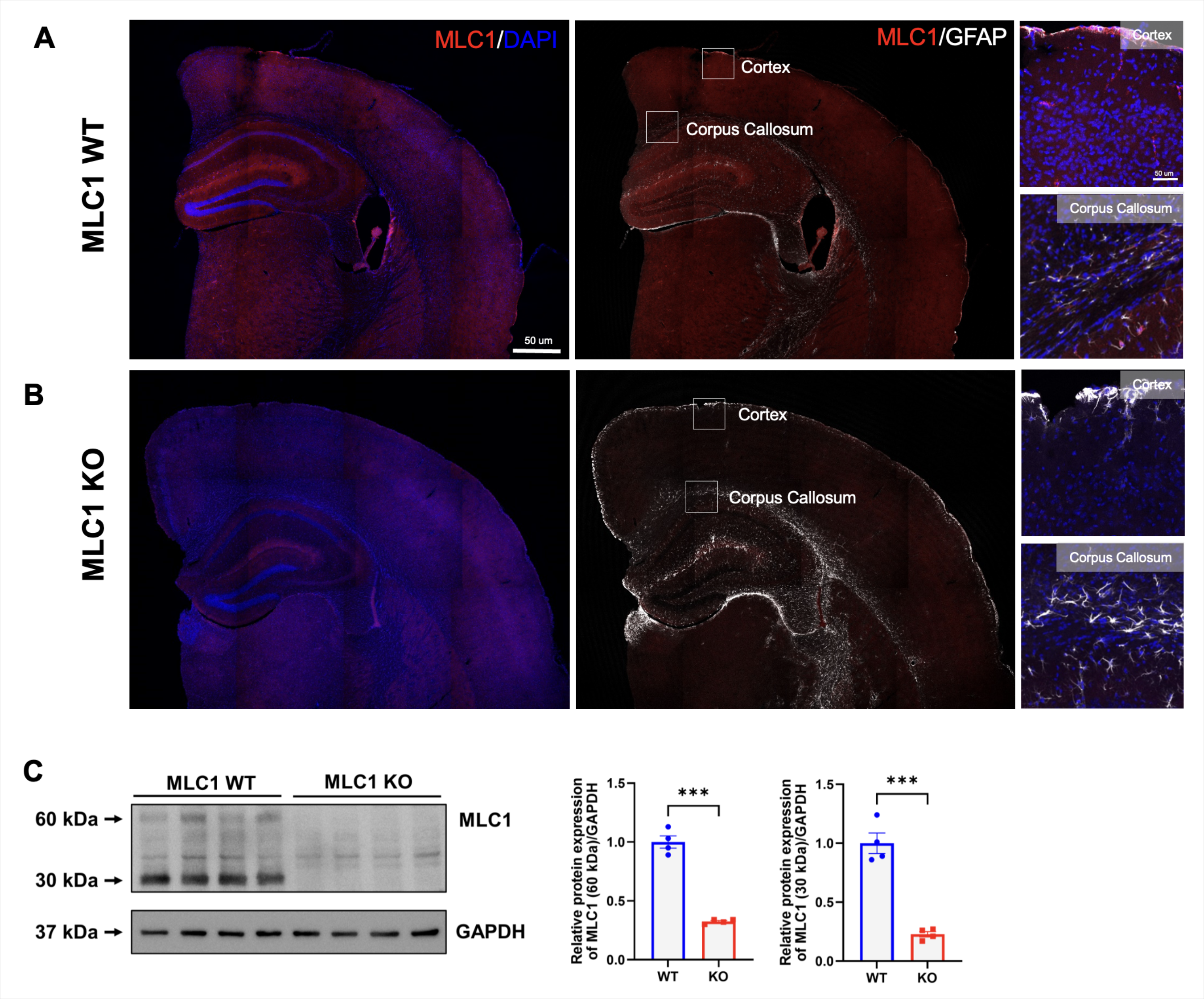

Supplement: Supplementary file 3 — Additional file 3: Figure S1. MLC1 immunohistochemistry and expression in astrocytes (A, B) of MLC1 WT and KO Mice. Regions indicated by white squares are magnified in the far-right column. MLC1 deletion is verified by western blotting, and MLC1 expression (C) is compared between MLC1 WT and KO mice. Three asterisks (***) indicate a P value smaller than 0.001. [file 12967_2023_4788_MOESM3_ESM.tiff]

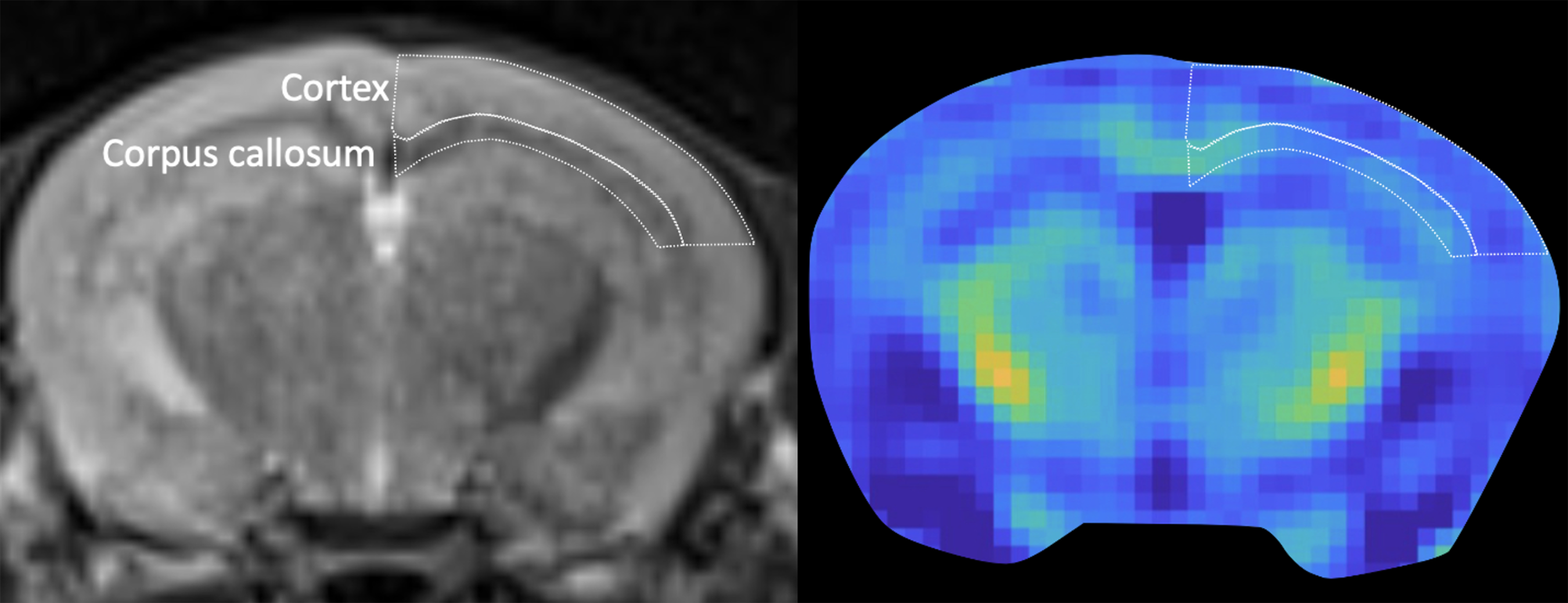

Supplement: Supplementary file 4 — Additional file 4: Figure S2. Regions of interest drawn in the cortex and corpus callosum in a mouse on the T2-weighted image (left) and myelin water fraction map (right). [file 12967_2023_4788_MOESM4_ESM.tiff]

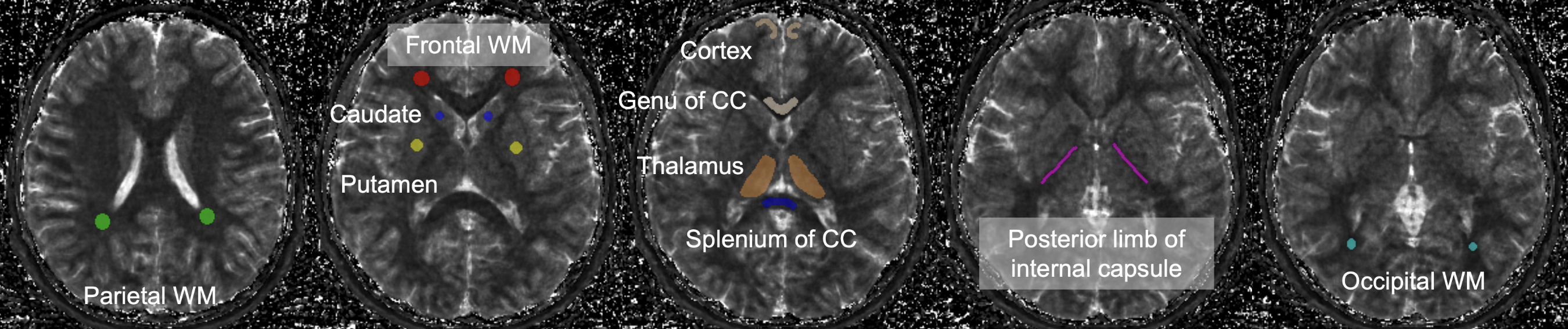

Supplement: Supplementary file 5 — Additional file 5: Figure S3. Regions of interest drawn in multiple brain regions in a child using T1 value map. WM = white matter, CC = corpus callosum. [file 12967_2023_4788_MOESM5_ESM.tiff]

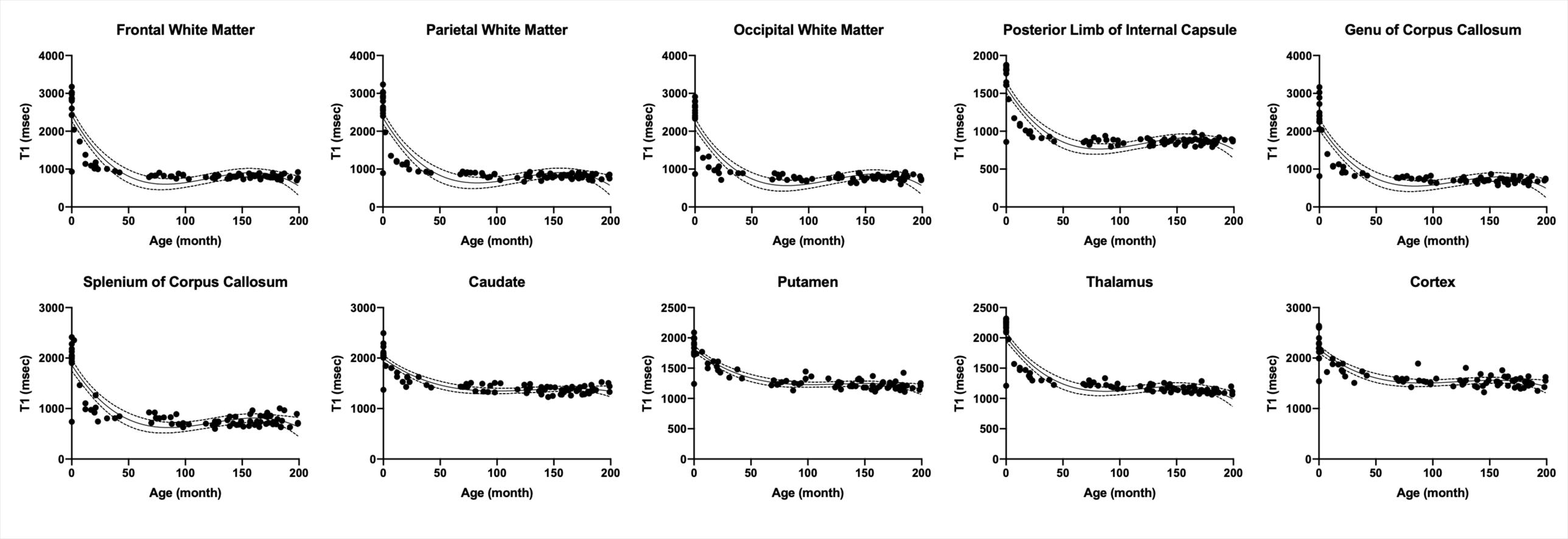

Supplement: Supplementary file 6 — Additional file 6: Figure S4. Scatter plots of T1 values in children according to age. Solid lines indicate the third-order regression lines of best fit, and dashed lines indicate the 95% confidence intervals. [file 12967_2023_4788_MOESM6_ESM.tiff]

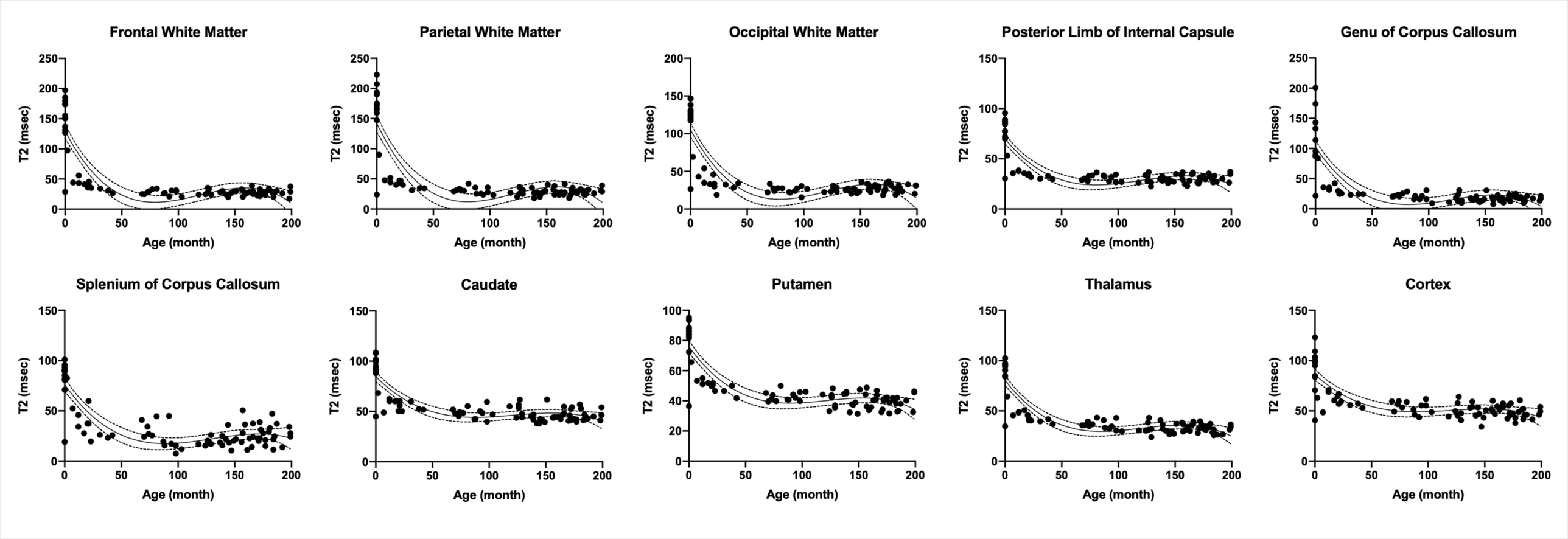

Supplement: Supplementary file 7 — Additional file 7: Figure S5. Scatter plots of T2 values in children according to age. Solid lines indicate the third-order regression lines of best fit, and dashed lines indicate the 95% confidence intervals. [file 12967_2023_4788_MOESM7_ESM.tiff]
